# Chiral Induced Spin Polarized Electron Current: Origin of the Chiral Induced Spin Selectivity Effect

**DOI:** 10.1021/acs.jpclett.5c00104

**Published:** 2025-04-24

**Authors:** Jonas Fransson

**Affiliations:** Department of Physics and Astronomy, Uppsala University, 75120 Uppsala, Sweden

## Abstract

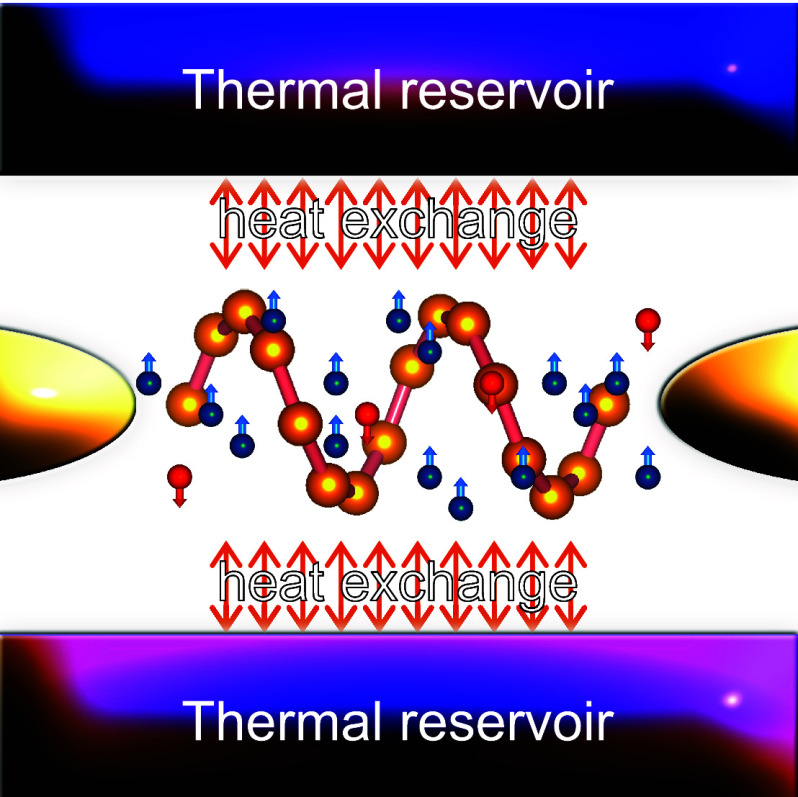

The discovery of the chiral induced spin selectivity effect has provided a novel tool to study how active physical and chemical mechanisms may differ in chiral enantiomers; however, the origin of the effect itself is yet an open question. In this Letter, it is theoretically shown that two aspects must be fulfilled for the chiral induced spin selectivity effect to arise. First, chirality is a necessary condition for breaking the spin-degeneracy in molecular structures that do not comprise heavy elements. Second, dissipation is indispensable for the molecule to develop a nonvanishing spin-polarization. These theoretical conclusions are illustrated in terms of a few examples, showing the necessity of the two aspects to be coordinated for the emergence of the chiral induced spin selectivity effect.

Chirality induced spin selectivity is now a well-established phenomenon in physics, chemistry, and biology.^1^ Whenever electrons flow through a chiral material, they will likely be spin-polarized in the direction they propagate.^2^ Aside from its intrinsic interest, this phenomenon has implications both for devices and for our understanding of biological electrochemistry. In aerobes large electron currents, typically tens of amperes in the resting human, flow from metabolism to oxygen.^3^ What makes spin important in this process of respiration is that the ultimate electron acceptor, dioxygen, is a ground-state triplet. One of the reactions involved in the reduction of oxygen to water involves a two-electron transfer. It has been shown that this reaction is facilitated by spin-polarization.^4^ At a ferromagnetic electrode, oxygen reduction proceeds faster in a magnetic field. Remarkably, general anesthetics, known to affect cellular respiration, markedly reduce spin-polarization at a ferromagnetic electrode.^5^ This said, the extent and importance of spin-polarization in living systems remains unknown. Direct two-terminal measurements of electron current in biology are usually impossible.

Hitherto, almost all measurements related to the chiral induced spin selectivity effect have thus far been made by injecting a spin-polarized current into the chiral material, e.g., see refs (6−8). In the case of photoemission, measurements using linearly polarized light were conducted,^9^ demonstrating the spin-polarization of the electrons emitted from a surface of chiral molecules. By contrast, there has been little attempt to discern the magnetic properties of chiral structures in the absence of external magnetic forces. If that were possible, it may amount to a noninvasive method to assess, for example, whether the electron currents flowing in a living organism are spin-polarized. Recently, however, it was predicted that chiral molecules should acquire an inhomogeneous spin-distribution under a charge current flux.^10^

Theoretically, the chiral induced spin selectivity effect has been approached using the independent particle description,^11−16^ which does not capture the anisotropic current response of chiral molecules when, e.g., changing the external magnetic conditions, that underlies the phenomenon. Density functional theoretical approaches have also been employed,^17,18^ however, without successfully accounting for these basic features of the effect. These approaches fail because of the intrinsic absence of particle correlations that account for dissipation or losses in the system. It has subsequently been demonstrated that electron correlations are necessary to be included in order obtain a somewhat more physically correct description of the processes.^19−29^ Specifically, formulating the physics pertaining to the chiral induced spin selectivity effect beyond the Born–Oppenheimer approximation has been outlined using different approaches, such as electron–phonon interactions^21−23,25,28,30^ and the impact of Berry forces.^31−33^ Despite the seemingly apparent coupling between electron–phonon interactions and dissipation, very little attention has been paid to its importance for the mechanism of spin selectivity in chiral structures. Furthermore, while dissipation was previously never considered crucial and, therefore, frequently not incorporated in modeling for spin selectivity, it has yet been discussed in several works.^12,21−23,25,28,30,34−40^ Only a few attempts have been outlined in which dissipative effects have been included in the modeling, also showing the importance for including this in the description.^21−23,25,35−37^

The prediction made in this Letter is that any charge current flowing through a chiral molecule must become spin-polarized. Behind the generation of the spin-polarized current are the breaking of time-reversal symmetry by dissipative processes and the breaking of spin-degeneracy by chirality coupled to spin–orbit coupling. In this sense, one may describe chiral molecules as being spin-polarizers. The mechanism presented in this Letter plays an important role in the theoretical development of the chiral induced spin selectivity effect.

Spin-polarization will emerge if two symmetries are broken, namely, (i) time-reversal symmetry and (ii) spin-degeneracy. It is not necessary for a single mechanism to break the two symmetries simultaneously. There might be different sources for the two symmetry breaking agents. One should keep in mind that breaking time-reversal symmetry allows but does not imply breaking spin-degeneracy. Another important aspect is that the discussion of breaking time-reversal symmetry does not necessarily apply to the system as a whole but only to parts that locally may correspond to lowering of the entropy as a local order is established. This loss of entropy is, however, compensated for by an excess of entropy in another part of the system. Consequently, the description of local properties may display a broken symmetry state, although the global symmetry is preserved.

An example is the screening that occurs around a localized magnetic moment in a metallic environment at low temperatures due to the Kondo effect. While the local moment tends to stabilize at low temperatures, the surrounding environment of itinerant electrons align antiferromagnetically with the local moment such that the overall magnetic moment vanishes. In this sense, the local order that is established by the formation of the localized moment is prevented from violating the time-reversal invariance by the Kondo screening cloud. It may be noted that the temperature under which the Kondo effect becomes viable may be high.^41^

The general structure of the electronic spin–orbit coupling can be effectively written as **v**·**σ**, where the vector **v** is defined by the electric field **E** and the momentum operator **p** (**v** ∼ **E** × **p** – **p** × **E**), and **σ** is the vector of Pauli matrices. For centrally symmetric (wave-) functions, the electric field **E** and, hence, the spin–orbit coupling vanishes. The wave function is required to be distorted from a centrally symmetric form to generate a spin–orbit coupling.

Consider a molecular structure defined by a distribution of coplanar nuclei. The effective spin–orbit coupling for such a structure provides a hybridization between the electron spins of planar form **v**·**σ** = *v*_–_σ_+_ + *v*_+_σ_–_, where *v*_±_ = *v*_*x*_ ± *iv*_*y*_ and σ_±_ = σ_*x*_ ± *iσ*_*y*_. For a Kramers doublet, the planar spin–orbit coupling breaks the degeneracy of the state, however, into bonding and antibonding linear combinations of the spin-states such that the resulting gapped electron spectrum remains non spin-polarized. In principle this symmetry breaking mechanism does not have an influence on the spin-degeneracy, which remains intact.

By contrast, for a noncoplanar distribution of nuclei, the effective vector field may include a longitudinal component *v*_*z*_. This leads to the introduction of the mass term *v*_*z*_σ_*z*_ in the model for the electronic structure. The significance of this mass term is to open up an energy gap between the spin-states, hence breaking the spin-degeneracy. It is important to point out that although this spin-degeneracy breaking mechanism exists in a molecule, it is an immanent property of the molecule. As such, it requires additional conditions to be fulfilled before an actual spin-polarization develops. Nevertheless, for chiral molecules, this mechanism has the effect to enable the emergence of a molecular spin-polarization under certain conditions that will be discussed next.

The most important of those conditions is breaking the time-reversal symmetry. Assume that the electronic structure of a molecular compound is captured by the single-electron Green function . Assuming that the Green function can be expressed in closed form as a Dyson equation, , where  and **Σ** represent the unperturbed Green function and self-energy, respectively. This is done in the paradigm in which the model Hamiltonian can be partitioned into noninteracting and interacting components. In such a representation, the density of electron states, DOS, is generally defined according to
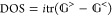
1where the lesser and greater forms  are proportional to the density of occupied and unoccupied electrons states, respectively. However, the density of electron states can equally well be formulated in terms of the retarded and advanced forms , thanks to the fundamental equality . Then, the Dyson equation allows to reformulate eq 1 as
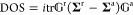
2since .

Keep in mind that the Green function can be written as the 2*N* × 2*N*-matrix , where the element  is a 2 × 2-matrix representing the electron propagation between states *m* and *n*, captured by the spinors ψ_*m*_ and ψ_*n*_^†^. In terms of the these 2 × 2 matrices, the expression can be further expanded as
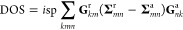
3where sp denotes the trace over spin 1/2 space.

Relating this expression to time-reversal symmetry is done by studying its properties under the time-reversal operator , where *K* denotes complex conjugation. Under the trace, the Pauli matrix σ^*y*^ commutes with the summand in eq 3; however, the complex conjugation in general does not, since *K*(Σ_*mn*_^r^ – Σ_*mn*_^a^) = −(Σ_*nm*_^r^ – Σ_*nm*_^a^)*K*. This expression is nonzero whenever the self-energy comprises an imaginary part. Since the imaginary part of the self-energy corresponds to dissipative processes in the system, the physical implication of this statement is that dissipation is a manifestation of broken time-reversal symmetry.

Note here that the discussion of the broken time-reversal symmetry has to do with the subsystem to which special attention is paid. The full system, which is a closed entity, is always time-reversal symmetric. However, the full properties of the subsystem are determined not only by its intrinsic configuration but also by the environment with which it interacts and exchanges, e.g., energy, momentum, and angular momentum. In these interactions, losses that are lost to the environment are inevitable.

The combination of mechanisms that break time-reversal symmetry and spin-degeneracy leads to an induced spin-polarization in chiral molecules. This conclusion can be demonstrated through a model of a vibrating molecule attached to metallic leads and embedded in a thermal reservoir, represented by the Hamiltonian
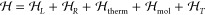
Here,  defines free electrons at the energy *ε*_**k**_ and wave vector **k** in the lead χ = *L*, *R* in terms of the spinor . The thermal reservoir is defined by the harmonic oscillators , where *b*_**q**_ and *b*_**q**_^†^ annihilates and creates a thermal phonon at the energy ω_**q**_ and wave vector **q**. The vibrating molecule is described by^23^
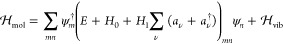
4a
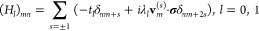
4band (*E*)_*mn*_ = δ_*mn*_ε_*m*_. The electrons in the molecule are distributed among  sites at the on-site energies ε_*m*_ and elastically hybridized with the nearest and next-nearest neighbors with rates *t*_0_ and λ_0_, as well as vibrationally assisted nearest and next-nearest hybridization with rates *t*_1_ and λ_1_. The next-nearest hybridization comprises the spin–orbit coupling **v**_*m*_^(*s*)^·σ, where , **d**_*m*,*s*_ = **r**_*m*_ – **r**_*m*+*s*_, and . The vector **v**_*m*_^(*s*)^ defines the curvature between the sites **r**_*m*_, **r**_*m*+*s*_, and **r**_*m*+2*s*_. The nuclear vibrations are represented by harmonic oscillations in = , which are annihilated and created by the phonon operator *a*_ν_ and *a*_ν_^†^, respectively, at the vibrational energy ω_ν_. The second term accounts for anharmonic contributions where Φ_*κμν*_ denotes the coupling strength and the operator *Q*_κ_ = *a*_κ_ + *a*_κ_^†^ represents the nuclear displacement.

Finally, the molecule is coupled to the leads and thermal reservoir through
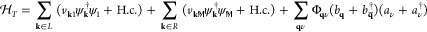
5where *v*_**k***m*_, , is the tunneling rate for electrons between the molecule and the leads, whereas Φ_**q**ν_ denotes the coupling rate between the nuclear vibrations and thermal phonons, and **q̅** = −**q**.

Here it should be remarked that the inclusion of the thermal reservoir  and its coupling to the nuclear vibrations, last term in eq 5, was never made explicit previously, see for instance refs (7, 23, and 42). However, the effect of the coupling to the thermal reservoir was implicitly included through the phonon lifetime τ_ph_, see eq 6b. In the light of the present model, this lifetime is directly associated with the presence of the thermal reservoir through the self-energy to the phonon propagator emerging from the coupling to this reservoir.

From this model, the electronic structure can be calculated in terms of the 2 × 2-matrix single-electron Green function **G**_*mn*_, which describes the electron propagation between sites *m* and *n*. To the second order in the vibrationally assisted hybridizations *t*_1_ and λ_1_, the Green function  for the whole molecule is given by^23^
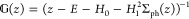
6a
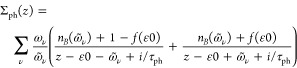
6bwhere . Here, the degrees of freedom associated with the leads are integrated out and captured by the addition of the level broadening Γ_χ_/2 to the energies , where Γ_χ_ = 2*π∑*_**k**∈χ_|*v*_**k***m*_|^2^δ(ω – *ε*_**k***m*_).

In this expression, the effects of the anharmonicity are included through the renormalized vibrational energy , where α_ν_ = – 36Φ^2^ coth(*βω*_ν_/2). Here, it is assumed that the coupling Φ_*κμν*_ = Φ for all modes. This renormalization arises from a second-order expansion of phonon propagator *D*_ν_(*z*) = ⟨⟨*Q*_ν_|*Q*_ν_⟩⟩(*z*) with respect to the anharmonic corrections (coupling Φ). Furthermore, the lifetime τ_ph_ arises from the coupling between the nuclear vibrations and thermal reservoir and explicitly accounts for the losses associated with inelastic processes. The lifetime is defined by , where *d*_**q**_^r^(ω) = 2ω_**q**_/[(ω + *iδ*)^2^ – ω_**q**_^2^], δ > 0 infinitesimal, is the unperturbed retarded propagator for phonons in the thermal reservoir. The lifetime is assumed to be structureless, which is justified for the thermal environment and a phonon bandwidth on the order of 1 eV.

The physical quantities of interest in the article are calculated from the lesser and greater forms  of the Green function. These forms are, here, given by the expressions , where **γ**_χ_ is the -matrix for the coupling to the left (χ = *L*) and right (χ = *R*) metal, whereas *f*_χ_(ω) = *f*(ω – μ_χ_) define the Fermi–Dirac distribution function at the chemical potential μ_χ_. The Green functions are normalized such that  = 1. In this way, for instance, the occupation number *n*_*m*_ at site *m* is obtained by calculating the integral *n*_*m*_ = (−*i*)sp∫**G**_*mm*_^<^(ω)*dω*/2π, whereas the corresponding spin projection ⟨**S**_*m*_⟩ = (−*i*)spσ∫**G**_*mm*_^<^(ω)*dω*/4π, such that, e.g., *n*_*m*↑_ = *n*_*m*_ + ⟨*S*_*m*_^*z*^⟩ and *n*_*m*↓_ = *n*_*m*_ – ⟨*S*_*m*_^*z*^⟩.

The parameters chosen for the calculations are typical for many molecular junctions, and especially the couplings to the phonons, *t*_1_ = *t*_0_/10 and λ_1_ = λ_0_/10, put the model in the weak coupling regime with respect to the electron–phonon interaction. Here, the elastic variables are set to *t*_0_ = 100 meV^43,44^ and λ_0_ = *t*_0_/25,^15,45^ whereas the coupling to the leads Γ_χ_ = *t*_0_/2 is a moderate and experimentally accessible strength. Then, with ε_*m*_ = – 5 eV, the full spectrum, including the inelastic effects, spans over about 9 eV centered around −5 eV, leaving a gap to the equilibrium chemical potential of about 0.5 eV. Changing the ratio between, e.g., *t*_0_ and λ_0_, changes the results quantitively, as one would expect, however, maintaining the qualitative aspects of the discussion.

Numerical results by applying the model to chiral and achiral molecules are shown in Figures 1 and 2 for structures schematically illustrated in the insets of Figure 1a,b. The achiral structure is a planar zigzag chain, Figure 1b, whereas the chiral deviates from planarity in one site that is noncoplanar with the zigzag structure, Figure 1a. The plots in Figure 1a,b show the site and spin-resolved charge distributions *n*_*mσ*_, , σ = *↑*, *↓*, for the (a) chiral and (b) achiral structure. The total site-resolved charge distributions *n*_*m↑*_ + *n*_*m↓*_ for both the chiral and achiral molecules are plotted in Figure 1c, whereas the corresponding site-resolved spin-polarization *n*_*m↑*_ – *n*_*m↓*_ is shown in Figure 1d. As predicted from the theoretical discussion above, the internal spin-degeneracy is broken in the chiral structure; see Figure 1a,d. The plots in Figure 1d, moreover, illustrate the opposite spin-polarizations arising in the two enantiomers. For the achiral structure, on the other hand, the charge distributions for the two spin-projections are equal, Figure 1b, which implies a vanishing spin-polarization, Figure 1d. As expected, the charge distributions for the chiral and achiral structures equal one another; see Figure 1c. In both structures, holding a total of 32 electrons, there is an excess charge near the interfaces to the metals rendering a slight charge depletion in the interior of the molecule.

**Figure 1:**
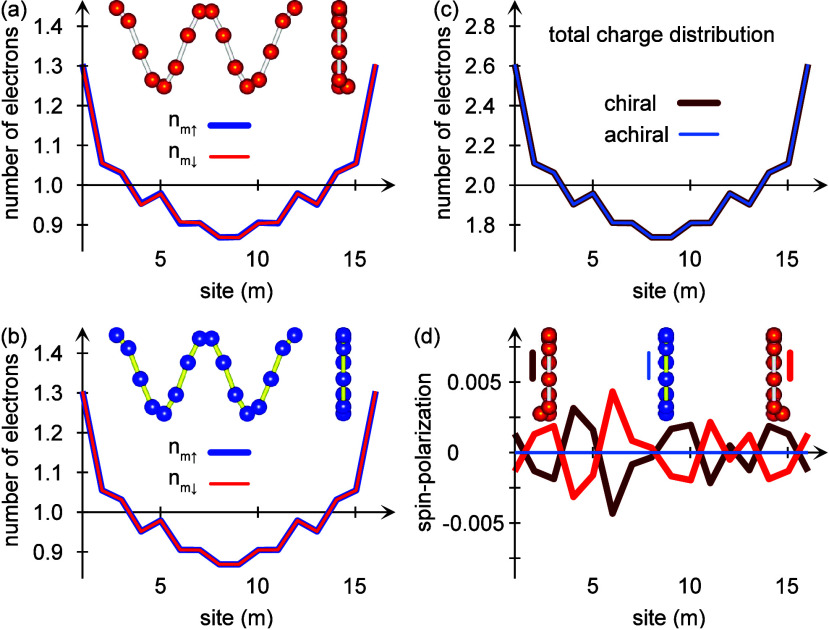
Calculated equilibrium site and spin-resolved charge distributions for (a) a chiral and (b) an achiral molecular chain comprising  sites. The total charge distributions *n*_σ_, σ = *↑*, *↓*, for both cases are shown in panel c, whereas the corresponding spin-polarizations *n*_*↑*_ – *n*_*↓*_ are plotted in panel d, where the spin-polarization for the opposite enantiomer is also shown. Here, ε_*m*_ = – 50, *t*_1_ = 1/10, λ_0_ = 1/25, λ_1_ = 1/250, Γ_χ_ = 1/2, and ω_ν_ = 1/50, in units of *t*_0_ = 0.1 eV, at the temperature *T* = 300 K.

**Figure 2:**
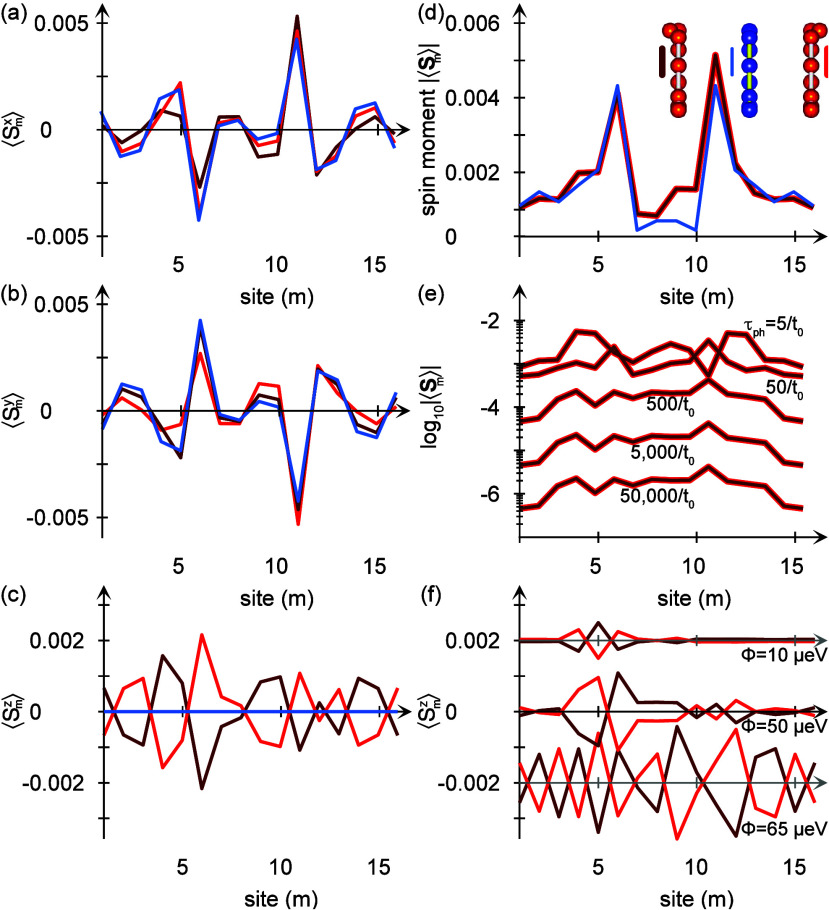
Calculated equilibrium site resolved spin-distributions projected in (a) ⟨*S*_*m*_^*x*^⟩, (b) ⟨*S*_*m*_^*y*^⟩, (c) ⟨*S*_*m*_^*z*^⟩, and (d) |⟨**S**_*m*_⟩| for an (blue) achiral and (red, dark red) two-enantiomers of chiral molecular chains comprising  sites. (e and f) The induced total spin moment |⟨**S**_*m*_⟩| for (e) increasing lifetime τ_ph_ and (f) increasing coupling Φ. Other parameters are as in Figure 1.

However, despite the absence of a spin-polarization in the achiral structure, there is nonetheless a nontrivial spin-texture, which can be seen in Figure 2a–c, in which the expectation value of the site-resolved spin-projections ⟨*S*_*m*_^α^⟩, α = *x*, *y*, *z* are plotted, as well as the total spin moment |⟨**S**_*m*_⟩|, Figure 2d. While the longitudinal spin-projection ⟨*S*_*m*_^*z*^⟩ = (*n*_*m*↑_ – *n*_*m*↓_)/2 vanishes, the spin–orbit coupling induces a transverse spin-texture ⟨*S*_*m*_^*x*,*y*^⟩ ≠ 0. The origin of this texture can be found in the curvature vector **v**_*m*_^(*s*)^ which is necessarily perpendicular to the longitudinal spin orientation σ^*z*^. However, for noncollinear achiral structures, the curvature vector **v**_*m*_^(*s*)^ is coplanar with σ^*x*,*y*^.

By contrast, in chiral structures the curvature vector **v**_*m*_^(*s*)^ also comprises a component in the longitudinal direction that breaks the chiral symmetry of the structure. Therefore, since the orbital degree of freedom is coupled to the spin through the spin–orbit coupling, the chiral symmetry breaking also breaks the spin-degeneracy, which can be seen in Figure 2. It is, moreover, important to notice that helicity is not necessary in this context, but the more general character chirality is what makes the difference. This is also known from experimental observations of the chiral induced spin selectivity effect on, e.g., cysteine and similar compounds.^4^

The total induced spin moment |⟨**S**_*m*_⟩| is plotted in Figure 2e, for different vibrational lifetime τ_ph_. The dependence on τ_ph_ illustrates the importance of dissipative processes for the induced spin-polarization in this context. The magnitude of the spin-moment decreases nearly an order of magnitude for each order of magnitude increase in the vibrational lifetime. This clearly indicates that (i) the chirality broken spin-degeneracy does not lead to a spin-polarization in a molecule where the nuclear vibrations are not directly coupled to the surroundings, such that (ii) the thermal environment has to be included in a full description of the chiral induced spin selectivity effect.

The total induced spin moment is, moreover, plotted in Figure 2f for increasing coupling Φ. These plots illustrate the qualitative differences one may expect for high versus low energies. While the low-energy vibrations, which correspond to a high degree of anharmonicity, tend to distribute the induced spin-moment throughout the molecule, high-energy, or fast, vibrations have a stronger localization effect on the spin-moment. This may be understood considering that the vibrational energy is associated with a time-scale, such that low vibrational energies allow more time for the electronic structure to adjust to its changing environment, hence, the spin-polarization may leak out to a larger extent of the molecule. By the same token, for higher energies there is less time for the electronic structure to adjust and therefore only the electronic and spin structure spatially close to the origin of the broken spin-degeneracy is significantly modified. Further away from the breaking point, the spin structure is altered by a smaller degree.

That there is an induced spin-polarization in chiral structures in equilibrium is a prerequisite for chiral induced spin-selectivity to arise. In the absence of such intrinsic spin anisotropy, the chiral enantiomers cannot respond anisotropically to an injected spin-polarized current. Effectively, these statements can be interpreted as that the molecule, when it is attached to one or more metals and, hence, acquires a spin-polarization ordered in a specific direction, exhibits a predefined enantiospecific spin preference for electron exchange with the metal(s). In the isolated state, the molecule is a closed shell system without any spin-order. By attaching the molecule to the metal, the closed shell nature of the molecule is broken since it should be considered as an open system when electrons may be exchanged between the metal and the molecule. However, because of the predefined spin preference in the heterogeneous metal–molecule system, the exchange rate depends on the spin of the electron—electrons with spin *↑* and *↓* enter and exit the molecule with different probabilities. This difference in probability is, then, either further enhanced or diminished by interfacing the molecule with a ferromagnet since the predefined spin-polarizations of the two subsystems will either act in concert or compete with each other. From this reasoning, the anisotropic current response of the molecule when it is exposed to changes in the external magnetic conditions can be understood.

The transport properties of the achiral and chiral structures depicted in Figure 1 are plotted in Figure 3, in which the (a) charge current and (b) current spin-polarization are shown as a function of the voltage bias across the junction. The current gap for voltage biases between ±10 reflects the energetic distance between the equilibrium chemical potential and the nearest (occupied) molecular orbitals. Although that the charge currents are equal in the (gray) achiral and (red) chiral structures, the latter constitute a nonvanishing current spin-polarization, as expected; see Figure 3b. These plots also demonstrate that the current spin-polarization is enantiospecific, that is, mirror symmetric with respect to enantiomer L or D, and is of the order of several percent of the charge current. This is illustrated by the plots of the ratio (*J*_*↑*_ – *J*_*↓*_)/(*J*_*↑*_ + *J*_*↓*_) in Figure 3c.

**Figure 3:**
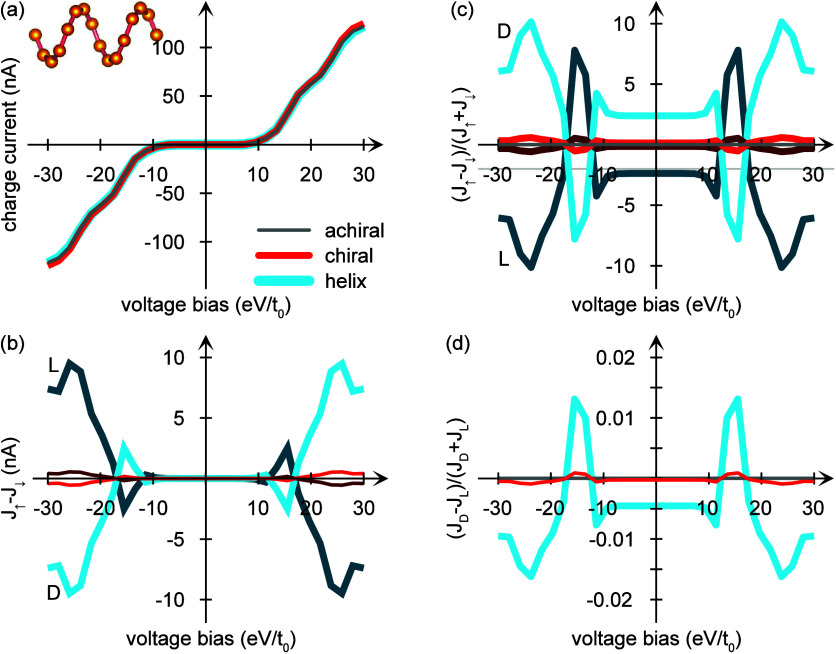
Transport properties for the (gray) achiral and (red) chiral structures depicted in Figure 1, as well as (blue) helical structures. The latter comprise  sites equidistantly distributed with respect to the azimuthal angle φ ∈ [0, 4π], as depicted in the inset of panel a. The panels show the (a) charge current, (b) current spin-polarization, (c) current spin-polarization normalized by the charge current, and (d) the enantiomeric asymmetry when injecting a 20% spin-polarized current from one of the leads. In panels b and c, the chiral enantiomers are assigned either *L* or *D*. In panel c, the faint line at −0.2 on the vertical axis represents zero spin-polarization for the conditions in panel d. Here, τ_ph_ = 5/*t*_0_ and Φ = 60 μeV. Other parameters are as in Figure 1.

The chiral structures depicted in Figure 1 are minimally chiral, in the sense that only one site is noncoplanar with all other sites. While this quality is sufficient to generate the spin-dependent properties observed for chiral structures, a comparison is made with a helical geometry in which the same number of sites (16) are equidistantly distributed with respect to the azimuthal angle φ ∈ [0, 4π], see inset of Figure 3a. The charge current (blue) of this structure is nearly the same as for the other two geometries; however, the current spin-polarization that arises is significantly stronger; see Figure 3b,c. Especially, as the voltage bias reaches the molecular orbitals and the current rises, it is accompanied by a strong spin-polarization from which it can be concluded that the molecular electronic structure needs to actively participate in the conduction through the molecule for a strong spin-polarization to arise. This is, however, obvious since gaps in the electronic structure are essentially vacuum which propagates electrons with exponentially small probability and, hence, cannot generate any significant spin-signatures.

The emergence of a current spin-polarization explains the results obtained in photoelectron spectroscopy experiments,^9^ where spin-polarized photoexcited currents were detected using Mott scattering. Detecting a spin-polarized intensity can only be the result of a spin-imbalance of the electrons that are ejected from the chiral molecules. In this experiment, electrons in the Au substrate are photoexcited and forced to be transported in the unoccupied orbitals of double-stranded DNA molecules. Applying the arguments used here for empty orbitals for a molecule attached to a single metal leads to the qualitatively same result as shows in Figure 3b,c. Likewise, since the spin-polarization is directly related with chirality of the used molecules, the results are enantiospecific, yielding opposite spin-polarizations for the two enantiomers, yet with equal amplitude.^42^

In the transport configuration, on the other hand, the chiral induced spin selectivity effect is quantified by, for instance, comparing the charge currents *J*_*D*_ and *J*_*L*_ measured for the *D* and *L* enantiomers under the injection of an externally spin-polarized current, respectively.^6^ The intrinsically generated chiral induced spin-polarization explains the phenomenon of the chiral induced spin selectivity effect in this setup, since there is an immanent spin anisotropy that can be enhanced by an externally injected spin-polarization, provided that the spin-polarizations are concurrent. If instead the spin-polarizations are competing, the molecular spin anisotropy is suppressed and eventually coerced to align with the external spin-polarization when this is sufficiently strong. The externally induced shift of the immanent spin-polarization is indicated by the faint line at −0.2 on the vertical axis in Figure 3c. Effectively, the current spin-polarization is shifted upward compared to zero spin-polarization when there is a 20% spin-polarization in one of the leads, pertaining to the spin-polarization obtained using magnetized Ni.^6^ In the calculation this is achieved by replacing, e.g., Γ_*L*_ → **γ**_*L*_ = Γ_*L*_(σ^0^ + *p*_*L*_σ^*z*^)/2, where *p*_*L*_ = 0.2 defines the spin-polarization of the magnetized lead.

Then, by comparison of the resulting charge currents for the two enantiomers through the ratio (*J*_*D*_ – *J*_*L*_)/(*J*_*D*_ + *J*_*L*_), the results for the chiral (red) and helical (blue) structures are plotted in Figure 3d. Both the weakly chiral and the helical structures display this enantiomeric asymmetry which has become a hallmark for the chiral induced spin selectivity effect in the transport configuration. The weakly chiral structure also has a weaker enantiomeric asymmetry than the helical, which can be understood as a large phase accumulation in the helical structure due to its repeated curvature property.

It is important to remark that the chiral induced spin-polarization and the chiral induced spin selectivity effect do not depend on the origin of the interactions and dissipation in the system. Replacing the vibrational and phonon degrees of freedom by electrostatic Coulomb repulsion at each site in the molecular chain results in the analogous results (see ref (20) for details), summarized for a realistic example in Figure 4.

**Figure 4:**
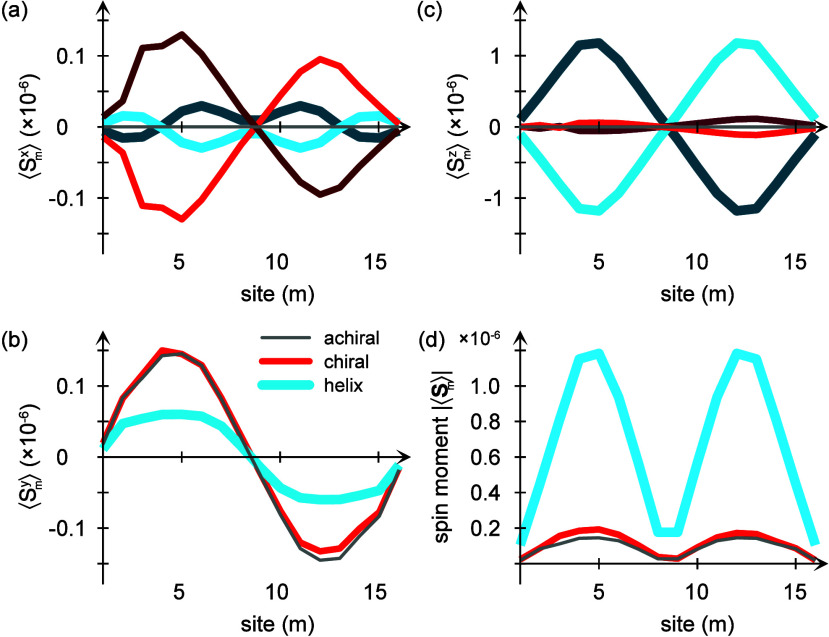
Calculated equilibrium spin-distributions projected in (a) ⟨*S*^*x*^⟩, (b) ⟨*S*^*y*^⟩, (c) ⟨*S*^*z*^⟩, and (d) |⟨**S**⟩| for an (blue) achiral and (red, dark red) two-enantiomers of chiral molecular chains comprising  sites. The plots represent calculations with on-site Coulomb repulsion. Here, ε_*m*_ = – 2, *U* = 1/5, λ_0_ = 1/100, and Γ_χ_ = 1, in units of *t*_0_ = 0.1 eV, at the temperature *T* = 300 K.

Similarly as in the example with vibrations, the achiral structure (gray) acquires a nontrivial spin-texture, Figure 4b and total spin moment Figure 4d. Nevertheless, there is no longitudinal spin component, Figure 4c. The chiral structures (red and blue), on the other hand, do acquire nonvanishing longitudinal spin components, Figure 4c,d, albeit small under the present conditions. The weak spin-polarization is related to the Coulomb repulsion in the present example being defined as a local on-site interaction only, whereas the vibrational effects in the previous example are nonlocal by construction. Hence, an addition of nonlocal Coulomb interactions would enhance the induced spin-polarization.^46^

It may be noticed that the parameters used in the calculations are within reasonable limits for typical molecules used in studies of the chiral induced spin selectivity effect. While the effective modeling may not be perceived as predictive when considering quantitative results, there is nevertheless a predictive capacity insofar as it provides a qualitative description of the phenomenology. Specifically, efforts have been made to maintain the bare spin–orbit coupling λ_0_ and λ_1_ orders of magnitude smaller than the hopping rate *t*_0_, since this is to be expected in organic molecules. It may, therefore, be concluded that electron correlations both enhance the effect of spin–orbit coupling and also generate the broken symmetry state, which is necessary for the chiral induced spin selectivity effect.

In conclusion, it has been demonstrated that dissipation in chiral structures leads to the formation of a nontrivial and inhomogeneous spin-density. Chirality is a manifestation of a structural broken symmetry, which by spin–orbit coupling breaks the spin-degeneracy. However, the formation of a spin-density requires a broken time-reversal symmetry, and since the formation of a spin-asymmetry, or spin-density, is also identical to the establishment of an order, the entropy of the electronic structure has to be lowered. One implication of this statement is that any structure that does not possess a mechanism for spin-dependent losses cannot maintain a spin-density as a steady state. A second implication is that the origin of the spin-dependent dissipative processes is of subordinate importance. Finally, one consequence of spin-dependent dissipation in chiral structures is the chiral induced spin selectivity effect.

## Supporting Information Available

Transparent Peer Review report available (PDF)
