# Supplementary material for: Chiral Induced Spin Polarized Electron Current: Origin of the Chiral Induced Spin Selectivity Effect
Source: J Phys Chem Lett. 2025 Apr 24;16(17):4346–53. doi: 10.1021/acs.jpclett.5c00104 (PMC12051198; doi:10.1021/acs.jpclett.5c00104)
Supplement: Supplementary file 1 — jz5c00104_si_001.pdf [file jz5c00104_si_001.pdf]

jz-2025-001048.R1

Name: Peer Review Information for "Chiral induced Spin Polarized Electron Current: Origin of the Chiral Induced Spin Selectivity Effect"

First Round of Reviewer Comments

Reviewer: 1

Comments to the Author

In his work "Chiral induced Spin Polarized Electron Current: Origin of the Chiral Induced Spin Selectivity Effect", Jonas Fransson reports a clear analysis of some conditions which must be fulfilled in order to observe a spin polarization in transport/photo-emission experiments through chiral systems. These are related to breaking both time-reversal symmetry (by spin orbit coupling related to the chiral structure of the molecule) and spin-degeneracy (by some form of dissipation). These general conclusions are supported by some examples, in which model chiral/achiral systems are compared. In particular, the Author considers a single-electron model with the explicit inclusion of a vibrational mode (which is in turn coupled to a thermal bath) and another one in which vibrations are replaced by electron-electron Coulomb repulsion.

Compared to previous works, I appreciate the efforts of the Author to give a clearer physical picture of the mechanisms driving the spin polarization. I think the paper represents an important step to set some key aspects to understand the phenomenon. Nonetheless, I still have some important questions which have to be addressed before recommending for publication:

- A general point: according to the general considerations in the first part of the paper, it seems that spin polarization can emerge even in a two-terminal device, provided time-reversal symmetry and spin degeneracy are removed. Is this correct or does the heat bath provide an effective additional terminal, as done in other approaches by using Buttiker virtual leads?

- Related to the previous one: an external (even weak) magnetic field would remove both time-reversal symmetry and spin-degeneracy. In this case, do you expect spin polarization even without dissipation?

- The Author should compare more directly with his previous works, in which the heat bath was not explicitly included (or at least not discussed, maybe due to some simple assumptions such as instantaneous thermalization of the vibrations).

- Minor point: the form of the SOC in Eq 4b is only for a helix or is it applied also to other geometries (such as the one in Fig 1a where chirality is not distributed over the molecule but point-like)? In this latter case a smaller spin polarization is found, but this is not always true. For instance, systems reported in [Science 382, 197 (2023)] or in [JACS 146, 24125 (2024)] are not helices, but show a sizable spin polarization, even larger than standard distributed helices such as DNA. Does the Author have any hint to effectively introduce this point chirality in simplified models (which do not rely on ab-initio calculations)?

- Concerning the investigated model systems, which is the number of electrons in the simulations? As far as I understood, an electron is propagated through an empty chain of orbitals. However, in Fig. 1 there seems to be about 1 electron per site (half filling). I think this should be clearly reported, also for the interacting case with the Coulomb repulsion.

- In Figs 1 and 2, it is not completely clear to me the meaning of “magnetic moment”, but maybe my lack of understanding is again related to the number of electrons per site. Is this the total spin of the whole molecule? In that case, it seems dissipation drives the system to the ground singlet. But then I do not understand its relationship with the site index. If it is a local spin, it should be normalized to the number of particles per site.

- The parameters in the simulations should be better discussed and justified. I understand that the spirit of the paper is to identify key aspects which can give non-zero polarization, but which is the meaning of a vibrational mode at 0.001 meV? In molecules, such a low frequency mode cannot exist. The lowest-energy optical modes (i.e. local vibrations) are observed at a few meV. This mode can only represent a rigid translation of a molecule within a crystal (an acoustic mode) but then how can it modulate anything at the molecular scale? Is it really necessary to use such a low-frequency mode? The Author should compare the current simulations with others using reasonable frequency modes.

- An important point recently discussed in the literature (see for instance works by Subotnik) is the need to overcome Born-Oppenheimer approximation, i.e. to consider spin-boson couplings which depend on the “moving” nuclear coordinates. Is it possible to account for this in the model?

• Finally, it would be important to further clarify the CISS mechanism at play. From the simulations of transport experiments, it seems the molecule acts as a spin filter. Can this be compared with photo-emission results? I would expect a different ejection rate for electrons if the enantiomer is changed. Is this correct?

Reviewer: 2

Comments to the Author

In this work, the prerequisites for chiral-induced spin selectivity are discussed, based on a Hamiltonian for a molecule coupled to leads that includes electron-phonon coupling and coupling to a thermal reservoir. These prerequisites for CISS are suggested to be structural chirality and dissipation. It is also suggested that the coupling and reservoir terms can be replaced by their electronic counterparts, in other words, that the specific nature of the physical mechanism through which these terms arise is not essential to produce CISS. This work builds on earlier work by the same author.

Studies like this one are important and needed urgently to shed light on the, as of now, unexplained mechanism underlying CISS. In that sense, it would be great to see this published in JPCL. However, in the present form, the work should not be published. Several issues are not clear and should be addressed before reconsidering the manuscript:

1) Overall, it looks like a similar model has been proposed before (by the same author), and the insight that chiral structures and dissipation are crucial to see CISS has been discussed in the community and in the literature for quite some time. To illustrate the merits of this work, it would be helpful to point out more clearly the new insights obtained in this work.

2) As mentioned, the importance of electron-phonon coupling and dissipation for CISS has been discussed in the literature before, by several authors including, e.g., Michael Thoss and Joseph Subotnik. Generally, the discussion of the existing literature is on the short side and should be extended to better place this work within the state of the art.

3) Without referring to earlier works by the author, it is hard to get a picture of what the model describes - presumably a chiral molecule connected to two (metal) leads, under bias voltage? Ir appears that the model is also meant to give insight into other experimental setups such as photoelectron CISS, but this is not clear from the Hamiltonian and the connection is only loosely discussed. It also remains unclear whether one of the leads is magnetic / if there is a magnetic electrode covered by a nonmagnetic metal such as gold (as in many experiments), whether spin-orbit coupling in the metal electrodes is important or not, which voltage ranges would be considered …

4) There is very little information on how the quantities plotted in Figures 1 through 4 were obtained besides the very brief "Numerical results by applying the model to chiral and achiral molecules are shown…" on page 8. I am not advocating for all details going into the main manuscript, but at least a Supplementary Material section with all information necessary to reproduce the results would be required.

5) It is mentioned which values were chosen for the various parameters in the model. What is missing is a) a physical justification for these values, and b), an analysis of how strongly the results would vary if those parameters were varied within reasonable physical limits. Along these lines, to what extent is the model predictive, i.e., would it be possible to predict the strength of CISS if an experimentalist approached a theorist with information on his or her chiral material and experimental setup?

6) Similarly, on page 11, a 20% spin-polarised current is assumed. This is likely motivated by approximate values for typical magnetic electrode materials such as nickel, but this remains unclear and should be clarified.

7) On page 5, it appears that a perturbative approach is employed, where interactions act as a perturbation. Which interaction(s) precisely, and how or why are the prerequisites fulfilled for them to be small enough for perturbation theory to hold?

8) The example given for local vs global symmetry breaking on page 4 (Kondo) is illustrative, yet it refers to a phenomenon that only occurs at low temperatures. CISS, by contrast, is

frequently observed at room temperature. Could the author provide examples that are more relevant to CISS in that they do not depend on cooling down the system?

9) The theoretical approach looks as if the vibrations/phonons were described as harmonic oscillators. This should be clarified. If this is the case, under which circumstances would one expect the harmonic approximation to hold, and can one safely assume that it will hold for the systems under study here? (in particular, for low-frequency vibrations, the harmonic approximation tends to do badly due to the large portion of the potential energy surface being sampled).

10) The term “chiral induced spin anisotropy” should be defined more clearly. In relation to this, on page 9, the statement “That there is an induced spin-polarization in chiral structures in equilibrium is a prerequisite for the chiral induced spin-selectivity to arise” should be discussed and justified in more detail. As it is, this being a prerequisite is not clear from the manuscript. It should also be discussed whether this suggested equilibrium spin polarisation in chiral structures is present intrinsically or whether it is tied to some current having flown through the chiral system at some point (e.g., when making contact with a surface).

11) It would be good to clarify the wording and notation in some instances, for example

- page 3: “discern the magnetic properties of chiral structures in the absence of magnetic boundary conditions” - what are magnetic boundary conditions in this context?

- page 4, “the vector v is defined by the electric field and the momentum operator p” - what does this mean mathematically?

- page 5: “hybridization between the spins of the planar form” - which spins precisely?

- page 5 “the matrix defining the spectrum” - which matrix, and which spectrum?

- page 6: What does the notation (G_nm)nm refer to? do the n/m indices refer to the same quantity in both instances, and if yes, why do they appear twice? What precisely are the states m and n discussed just below? and why are there apparently only two (resulting in 2x2 matrices)?

- page 9: “the orbital quality is coupled to the spin” - what is an orbital quality?

- page 9, middle: What is a “closed molecule” in the context of this work?

- page 10: “the gap between the orbitals, which essentially is vacuum” - the informed reader gets an idea of what may be intended with this statement, but the use of the term “vacuum” still appears misleading.

Author's Response to Peer Review Comments:

Institutionen för Fysik och Astronomi
Dr. Jonas Fransson
Professor

Besöksadress:
Ångströmlaboratoriet,
Polacksbacken
Lägerhyddvägen 5

Postadress:
Box 516
751 21 Uppsala

Telefon:
018 471 5864

Telefax:
018 471 3524

Mobil:
070 167 9264

Hemsida:
http://www.physics.uu.se

Epost:
Jonas.Fransson@physics.uu.se

Department of Physics and Astronomy
Jonas Fransson
Professor

Visiting address:
Ångströmlaboratoriet,
Polacksbacken
Lägerhyddvägen 5

Postal address:
Box 516
SE-751 21 Uppsala
SWEDEN

Telephone:
+46 18 471 5864

Telefax:
+46 18 471 3524

Cell:
+46 70 167 9264

Web page:
http://www.physics.uu.se

Email:
Jonas.Fransson@physics.uu.se

Dear Editor

I hereby resubmit my manuscript *Chiral induced Spin Polarized Electron Current: Origin of the Chiral Induced Spin Selectivity Effect*, for consideration for publication in the Journal of Physical Chemistry Letters.

The reviewers are both in favor of this article and have asked for a revision of the text for the sake of clarifying certain issues. Overall, the criticism the reviewers have are very constructive and helpful, and when reading their comments I can surely see the benefit, and necessity, for making a clearer presentation of the material. Below, I have responded to the raised issues in detail. All changes are high-lighted in the additional manuscript pdf.

In order to accommodate the critique raised by the reviewers, the article has been extensively revised leading to also extension of its length.

Having addressed the questions raised by the reviewers and made corresponding amendments in the manuscript, I believe that my article is suitable for publication in the Journal of Physical Chemistry Letters.

Yours sincerely,

Jonas Fransson

Response to the first reviewer

In his work "Chiral induced Spin Polarized Electron Current: Origin of the Chiral Induced Spin Selectivity Effect", Jonas Fransson reports a clear analysis of some conditions which must be fulfilled in order to observe a spin polarization in transport/photo-emission experiments through chiral systems. These are related to breaking both time-reversal symmetry (by spin orbit coupling related to the chiral structure of the molecule) and spin-degeneracy (by some form of dissipation). These general conclusions are supported by some examples, in which model chiral/achiral systems are compared. In particular, the Author considers a single-electron model with the explicit inclusion of a vibrational mode (which is in turn coupled to a thermal bath) and another one in which vibrations are replaced by electron-electron Coulomb repulsion. Compared to previous works, I appreciate the efforts of the Author to give a clearer physical picture of the mechanisms driving the spin polarization. I think the paper represents an important step to set some key aspects to understand the phenomenon. Nonetheless, I still have some important questions which have to be addressed before recommending for publication:

- **Reviewer comment 1)** *A general point: according to the general considerations in the first part of the paper, it seems that spin polarization can emerge even in a two-terminal device, provided time-reversal symmetry and spin degeneracy are removed. Is this correct or does the heat bath provide an effective additional terminal, as done in other approaches by using Buttiker virtual leads?*

  My response: It is correctly pointed out that the two-terminal device can spin-polarize, which is actually illustrated be the case at the end of the paper. The combination of Coulomb interaction, the non-Hermiticity introduced by the couplings to the leads, spin-orbit coupling, and chirality opens up for the emergence of the spin-polarization. In vibrational case, on the other hand, the vital non-Hermiticity which also enables leakage, is provided by the heat bath. In this case, then, the heat bath may be viewed as a concrete form of a Büttiker probe. In addition, the same coupling also opens up for a spin-polarization even in the single terminal case, as was discussed in Nano Lett. **21**, 3026 (2021).

- **Reviewer comment 2)** *Related to the previous one: an external (even weak) magnetic field would remove both time-reversal symmetry and spin-degeneracy. In this case, do you expect spin polarization even without dissipation?*

  My response: Certainly, should the molecular level degeneracy be broken by, for instance, a Zeeman splitting the resulting molecular electronic structure would be spin-polarized even without the heat bath. The spin-polarization would in this case scale with the strength of the external magnetic field.

- **Reviewer comment 3)** *The Author should compare more directly with his*

previous works, in which the heat bath was not explicitly included (or at least not discussed, maybe due to some simple assumptions such as instantaneous thermalization of the vibrations).

My response: This is a good point and I have added comments about the inclusion of the heat bath. As a matter of fact, the effect of the heat bath was included also in the previous publications, however, I did not realize by then its importance for the induced spin-polarization. While the broadening of the resonances in the self-energy was included I have not until now understood its physical origin and physical implications. However, inclusion of the heat bath makes the model more realistic since any system would be immersed in a thermal bath of some kind.

- **Reviewer comment 4)** *Minor point: the form of the SOC in Eq 4b is only for a helix or is it applied also to other geometries (such as the one in Fig 1a where chirality is not distributed over the molecule but point-like)? In this latter case a smaller spin polarization is found, but this is not always true. For instance, systems reported in [Science 382, 197 (2023)] or in [JACS 146, 24125 (2024)] are not helices, but show a sizable spin polarization, even larger than standard distributed helices such as DNA. Does the Author have any hint to effectively introduce this point chirality in simplified models (which do not rely on ab-initio calculations)?*

  My response: First, the same model as it is written around Eq. (4) is applied to the geometries considered in this paper. An obvious reason is that I want to discuss the role of the geometry for the different mechanisms to become activated. As can be seen in Fig. 2 (a), (b), also the achiral zig-zag structure acquires a spin-orbit coupling which results in the non-vanishing transverse spin-projections. However, as is discussed in depth in the paper, it is only in a chiral structure where the geometry also couples to the longitudinal spin-projection.

  Nonetheless, the model does not depend on the structure being helical, it applies to any geometry. It should be mentioned, though, that the helix is simple to code.

  Whether a non-helical chiral structure would lead to a stronger spin-polarization than for helical is something that I cannot discern from the references given by the reviewer. These references emphasize that it is not necessary to use helical structures in the context of the chiral induced spin-selectivity effect, something which is clearly important and valuable knowledge.

  The results shown here are the first calculated ones that demonstrate the connection between non-helical chirality and spin-polarization. Using the minor chirality as is done for the structure in, e.g., Fig. 1 (a), is made for that particular purpose – to demonstrate that minor deviations from an achiral structure rendering chirality suffice for the effect to emerge.

- **Reviewer comment 5)** *Concerning the investigated model systems, which is the number of electrons in the simulations? As far as I understood, an*

electron is propagated through an empty chain of orbitals. However, in Fig. 1 there seems to be about 1 electron per site (half filling). I think this should be clearly reported, also for the interacting case with the Coulomb repulsion.

My response: The number of electrons in the structure is always determined by the chemical potential. The calculations are based on the energy levels $\varepsilon_m = -5$ eV below the Fermi level of the metals. The band width of the calculated spectrum, using the parameters given in Fig. 1, is around 9 eV and the highest energy in the spectrum lies about 0.5 eV below the Fermi level of the metals. Hence, the molecule is essentially filled with electrons which is shown in Fig. 1 (c) – charge distribution shows in average two electrons per site.

It is correct that there is nothing said about this in the text and I thank the reviewer for raising this issue. I have added statements about the charge distribution in the discussion of Fig. 1. I have also added an inset of the charge distribution for the Coulomb repulsion set-up as well as added the corresponding text.

- **Reviewer comment 6)** *In Figs 1 and 2, it is not completely clear to me the meaning of "magnetic moment", but maybe my lack of understanding is again related to the number of electrons per site. Is this the total spin of the whole molecule? In that case, it seems dissipation drives the system to the ground singlet. But then I do not understand its relationship with the site index. If it is a local spin, it should be normalized to the number of particles per site.*

  My response: In Fig. 1 is the spatially resolved spin-polarization plotted, which is the difference $n_{m\uparrow} - n_{m\downarrow}$ for $m = 1, \ldots, \mathbb{M}$. In Fig. 2, the spatially resolved spin projections $\langle S_m^i \rangle$, $i = x, y, z$, are plotted, as well as the corresponding spin moment $|\langle \mathbf{S}_m \rangle| = \sqrt{\sum_i (S_m^i)^2}$. However, the notation I have used was quite ambiguous and I have clarified better the meaning of the symbols. Moreover, I have added text showing how the plotted quantities are defined in terms of the Green function in Eq. (6).

  In the plots of the spins, the values are normalized correctly with the number of totally allowed charge in the molecule, $2\mathbb{M}$.

- **Reviewer comment 7)** *The parameters in the simulations should be better discussed and justified. I understand that the spirit of the paper is to identify key aspects which can give non-zero polarization, but which is the meaning of a vibrational mode at 0.001 meV? In molecules, such a low frequency mode cannot exist. The lowest-energy optical modes (i.e. local vibrations) are observed at a few meV. This mode can only represent a rigid translation of a molecule within a crystal (an acoustic mode) but then how can it modulate anything at the molecular scale? Is it really necessary to use such a low-frequency mode? The Author should compare the current simulations with others using reasonable frequency modes.*

My response: The reviewer raises an important and delicate issue about the energy of the nuclear vibrations. Stimulated by the questions, I have added a second inset in Fig. 2, showing the dependence of the vibrational energy on the induced spin-moment, as well as an accompanying discussion. The lower the energy of the vibration, the more the induced spin-polarization becomes dispersed throughout the structure, while higher energy vibrations tend to localize the effect around the chiral center. There is a time-scale associated with the vibrational energy which suggests the interpretation that the lower the frequency, the more time to adjust to its surrounding is given to the electronic, hence also spin, structure, and vice versa. With such an interpretation one may understand that the larger vibrational energies tend to localize the spin moment around the chiral center which is the main source for emergence of spin-polarization.

Thanks to the issue raised by the reviewer, I have modified Fig. 2 and included a new panel, (f), showing the development of the spin moment $|\langle \mathbf{S}_m \rangle|$ of increasing vibrational energy, and accompanying discussion in the text.

- **Reviewer comment 8)** *An important point recently discussed in the literature (see for instance works by Subotnik) is the need to overcome Born-Oppenheimer approximation, i.e. to consider spin-boson couplings which depend on the "moving" nuclear coordinates. Is it possible to account for this in the model?*

  My response: I am glad that the reviewer raises this issues, both since I have failed to refer to the important discussion by Subotnik, and also to clarify the meaning of the electron phonon interaction. The motion of the nuclei is included via the phonon operators, particularly, through the operator $a_\nu + a_\nu^\dagger$ in the contribution $H_1 \sum_\nu (a_\nu + a_\nu^\dagger)$, Eq. (4), which represents the coupling between the electrons and the nuclear displacement. Inclusion of this contribution takes the model beyond the Born-Oppenheimer approximation. In the present context, the factor H_1, Eq. (4b), provides a connection between the electron spin and the nuclear motion.

- **Reviewer comment 9)** *Finally, it would be important to further clarify the CISS mechanism at play. From the simulations of transport experiments, it seems the molecule acts as a spin filter. Can this be compared with photo-emission results? I would expect a different ejection rate for electrons if the enantiomer is changed. Is this correct?*

  My response: Yes, I claim that the physics described in the paper is also relevant for the photo-emission results. As such, the spin-polarization changes sign when changing the enantiomer, similarly as is discussed both here and in Nano Lett. **21**, 3026 (2020). For the sake of clarity, I have added additional comments about the photo-emission and how it would be understood in the context of the results presented here.

Response to the second reviewer

In this work, the prerequisites for chiral-induced spin selectivity are discussed, based on a Hamiltonian for a molecule coupled to leads that includes electron-phonon coupling and coupling to a thermal reservoir. These prerequisites for CISS are suggested to be structural chirality and dissipation. It is also suggested that the coupling and reservoir terms can be replaced by their electronic counterparts, in other words, that the specific nature of the physical mechanism through which these terms arise is not essential to produce CISS. This work builds on earlier work by the same author.

Studies like this one are important and needed urgently to shed light on the, as of now, unexplained mechanism underlying CISS. In that sense, it would be great to see this published in JPCL. However, in the present form, the work should not be published. Several issues are not clear and should be addressed before reconsidering the manuscript:

- **Reviewer comment 1)** *Overall, it looks like a similar model has been proposed before (by the same author), and the insight that chiral structures and dissipation are crucial to see CISS has been discussed in the community and in the literature for quite some time. To illustrate the merits of this work, it would be helpful to point out more clearly the new insights obtained in this work.*

  My response: It is a valid point raised by the reviewer which I appreciate. The role of dissipation has not been very directly and explicitly discussed. To my knowledge it is only in Adv. Materials 2313708 (2024) where dissipation was explicitly brought up. However, the effect of phonon and molecular motion has been discussed, by myself and a few others. There is, nevertheless, reason to make an outline of the novelties discussed in the present paper. This has been added in the introductory part of the revised paper.

- **Reviewer comment 2)** *As mentioned, the importance of electron-phonon coupling and dissipation for CISS has been discussed in the literature before, by several authors including, e.g., Michael Thoss and Joseph Subotnik. Generally, the discussion of the existing literature is on the short side and should be extended to better place this work within the state of the art.*

  My response: It is correctly pointed out that the discussion of the existing literature is short. I have also missed to mention the works by Subotnik and Thoss, among a few additional, which now are added and included in the introductory part of the revised paper.

- **Reviewer comment 3)** *Without referring to earlier works by the author, it is hard to get a picture of what the model describes - presumably a chiral molecule connected to two (metal) leads, under bias voltage? Ir appears that the model is also meant to give insight into other experimental setups such as photoelectron CISS, but this is not clear from the Hamiltonian and*

the connection is only loosely discussed. It also remains unclear whether one of the leads is magnetic / if there is a magnetic electrode covered by a nonmagnetic metal such as gold (as in many experiments), whether spin-orbit coupling in the metal electrodes is important or not, which voltage ranges would be considered ...

My response: It is correct that there is not extensive information about the potential realization of the model calculations, however, those are used more as examples to underline the initial discussion of the paper, which is the more important discussion. However, above Eq. (4), it is clearly stated that the first examples pertain to a vibrating molecule attached to metallic leads and embedded in a thermal reservoir. The metals are certainly non-magnetic – it is only in connection with the discussion of Fig. 3 (c), (d) there is a mention of injecting (20 %) spin-polarized electrons, which is clearly stated as making the replacement of the coupling Γ_L to the left lead to $\mathbf{\Gamma}_L = \Gamma_L(\sigma^0 + p_L\sigma^z)/2$, which is a standard way to provide spin-polarization from the lead.

The purpose of the paper is, however, not to go through any possible mechanism that may or may not play a vital role for the chiral induced spin-selectivity effect. It is rather the crucial feature of dissipation, which breaks time-reversal symmetry, and spin-orbit coupling combined with chirality, that leads to breaking the spin-degeneracy, that is under discussion and the examples are provided as concrete demonstrations of the theoretical conclusion. The models used in this paper have both been discussed previously and are not the main scope of the paper.

- **Reviewer comment 4)** *There is very little information on how the quantities plotted in Figures 1 through 4 were obtained besides the very brief "Numerical results by applying the model to chiral and achiral molecules are shown..." on page 8. I am not advocating for all details going into the main manuscript, but at least a Supplementary Material section with all information necessary to reproduce the results would be required.*

  My response: I agree that there is not much detail about how the plotted quantities are obtained. I have, therefore, added more information about the relation between the plotted quantities and the Green function provided in Eq. (6). Nonetheless, I cannot add much more since the calculations are really just a matrix inversion, as the one given in Eq. (6). I have filled the gaps between Eq. (6) to the definition of the plotted quantities which should be sufficient for anyone to reproduce the results.

- **Reviewer comment 5)** *It is mentioned which values were chosen for the various parameters in the model. What is missing is a) a physical justification for these values, and b), an analysis of how strongly the results would vary if those parameters were varied within reasonable physical limits. Along these lines, to what extent is the model predictive, i.e., would it be possible to predict the strength of CISS if an experimentalist approached a theorist*

with information on his or her chiral material and experimental setup?

My response: It is correctly pointed out that I have no discussion of the sensitivity of the results on the input parameters. Again, while I have added a brief discussion about these aspects, the scope of the paper is not to map out the viable range of parameters. First, that would be a formidable task since the models depend on many parameters, and second, the intention with the paper is not show how large the effects may be. What I am demonstrating is that spin-dependent dissipation plays a vital role for making the calculated chiral induced spin-selectivity effect comparable with experiments in terms of order of magnitude – percents and not fractions of percents.

- **Reviewer comment 6)** *Similarly, on page 11, a 20% spin-polarised current is assumed. This is likely motivated by approximate values for typical magnetic electrode materials such as nickel, but this remains unclear and should be clarified.*

  My response: Yes, the 20 % is taken since it is a common spin-polarization provided from, e.g., Ni or Co electrodes. Larger spin-polarizations are typically not relevant when using standard metals. I agree, however, that this should be mentioned and referenced, which it is now in the revised paper.

- **Reviewer comment 7)** *On page 5, it appears that a perturbative approach is employed, where interactions act as a perturbation. Which interaction(s) precisely, and how or why are the prerequisites fulfilled for them to be small enough for perturbation theory to hold?*

  My response: It is correctly pointed out that the discussion is based on a perturbative approach. In the general discussion the reviewer is mentioning, there is no concrete interaction mentioned since the aim is to pull out from the logics what the interaction has to include in order to provide the desired result. The examples provided after the general discussion are, however, concrete manifestations of such perturbations. In the discussion, I assume that the Green function can be written as a Dyson equation, an assumption suggesting that the necessary conditions are met already.

- **Reviewer comment 8)** *The example given for local vs global symmetry breaking on page 4 (Kondo) is illustrative, yet it refers to a phenomenon that only occurs at low temperatures. CISS, by contrast, is frequently observed at room temperature. Could the author provide examples that are more relevant to CISS in that they do not depend on cooling down the system?*

  My response: I am pleased to see that the reviewer appreciates the example with the Kondo effect. I understand that it may appear off topic as far as the temperature is concerned, by considering it to appear only at very low temperatures. However, the Kondo effect itself does not require very low temperatures. There are systems in which the Kondo temperature is above room temperature, see, e.g., Appl. Phys. Lett. **110**, 222407 (2017), which would make the example relevant. Moreover, the analogy was about the screening

and whether it occurs at low temperature or not is of subordinate relevance here. For the sake of demonstrating the relevance concerning temperature, I have added reference to examples with high Kondo temperature.

- **Reviewer comment 9)** *The theoretical approach looks as if the vibrations/phonons were described as harmonic oscillators. This should be clarified. If this is the case, under which circumstances would one expect the harmonic approximation to hold, and can one safely assume that it will hold for the systems under study here? (in particular, for low-frequency vibrations, the harmonic approximation tends to do badly due to the large portion of the potential energy surface being sampled).*

  My response: The vibrations in the calculations are assumed to be harmonic. In a sense, this approximation represents the worst scenario for the spin-dependent electron-phonon coupling as anharmonic contributions will lead to an even stronger such coupling. That I am assuming harmonic oscillations is written in the text.

- **Reviewer comment 10)** *The term "chiral induced spin anisotropy" should be defined more clearly. In relation to this, on page 9, the statement "That there is an induced spin-polarization in chiral structures in equilibrium is a prerequisite for the chiral induced spin-selectivity to arise" should be discussed and justified in more detail. As it is, this being a prerequisite is not clear from the manuscript. It should also be discussed whether this suggested equilibrium spin polarisation in chiral structures is present intrinsically or whether it is tied to some current having flown through the chiral system at some point (e.g., when making contact with a surface).*

  My response: It is an important issue raised by the reviewer, since if the message I aim to communicate does not go through to the reader, the text needs to be revised. I have done so around the discussion pointed out by the reviewer and hope that the message is clearer.

- **Reviewer comment 11)** *It would be good to clarify the wording and notation in some instances, for example*

  - page 3: "discern the magnetic properties of chiral structures in the absence of magnetic boundary conditions" - what are magnetic boundary conditions in this context?

  - page 4, "the vector v is defined by the electric field and the momentum operator p" - what does this mean mathematically?

  - page 5: "hybridization between the spins of the planar form" - which spins precisely?

  - page 5 "the matrix defining the spectrum" - which matrix, and which spectrum?

  - page 6: What does the notation $(G_{nm})nm$ refer to? do the n/m indices refer to the same quantity in both instances, and if yes, why do they appear

twice? What precisely are the states m and n discussed just below? and why are there apparently only two (resulting in 2x2 matrices)?

- page 9: "the orbital quality is coupled to the spin" - what is an orbital quality?

- page 9, middle: What is a "closed molecule" in the context of this work?

- page 10: "the gap between the orbitals, which essentially is vacuum" - the informed reader gets an idea of what may be intended with this statement, but the use of the term "vacuum" still appears misleading.

My response: I thank the reviewer for pointing out inaccuracies in the text. I have made amendments to these and hope that the text has become clearer.

Here I comment on the notation $\mathbb{G} = \{\mathbf{G}_{mn}\}_{mn}$, $m, n \in \{1, 2, \ldots, N\}$, which refers to a $N \times N$-matrix in which each entry itself if a 2×2-matrix Green functions $\mathbf{G}_{mn}(t, t') = (-i)\langle \mathrm{T}\psi_m(t)\psi_n(t')\rangle$ in terms of the spinor $\psi_m = (\psi_{m\uparrow}\ \psi_{m\downarrow})^t$. The Green function $\mathbf{G}_{mn}(t, t')$ denotes the propagation of a particle (electron) between the mth and nth states represented by ψ_m and $\psi_n^\dagger$ at the times t and t', respectively. The Green function $\mathbb{G}$ represents the propagation for all electrons captured by the $2N \times 2N$ states, where the factor 2 is included to account for spin. There is a 2×2-matrix Green function associated for each combination of states m, n, because of the spin-degree of freedom. I agree that I should have been clearer with the usage of notation and have amended this in the revised paper.

jz-2025-001048.R2

Name: Peer Review Information for "Chiral induced Spin Polarized Electron Current: Origin of the Chiral Induced Spin Selectivity Effect"

Second Round of Reviewer Comments

Reviewer: 1

Comments to the Author

The Author answered most of my questions in the revised manuscript.

However, one point has not still been satisfactorily addressed. The frequency of the vibrational modes used in most of the simulations is unreasonably small for real molecules. Such low energy vibrations can only exist in the solid state (or on a surface) and correspond to long wavelength acoustic phonons, i.e. rigid translations of the molecule. Hence, they cannot influence significantly Hamiltonian parameters at the molecular scale such as the nearest-neighbor hopping. In general, I would expect low-energy modes to have a very small electron-phonon coupling.

Hence, before recommending publication, the Author should include simulations of the spin polarization and of the current (Figs. 1 and 3) with a reasonable frequency of the vibrations in the meV range and explicitly report in the main text this parameter.

Reviewer: 2

Comments to the Author

The manuscript has been improved. There are still open questions and issues, in particular concerning the novelty of this work compared to previous discussions in the literature and the range of validity of the proposed model:

- Comment on reply to my comment 1):

The (potential) importance of dissipation in CISS was, contrary to what the author is stating, brought up and discussed as an essential component for the CISS mechanism in several instances in the literature, e.g.:

https://doi.org/10.1002/adma.202106629

https://doi.org/10.1021/acs.jpclett.0c00474

https://doi.org/10.1103/PhysRevB.104.024430

https://doi.org/10.1021/acs.jpclett.8b02196

https://doi.org/10.1021/acs.jpclett.3c02546

https://doi.org/10.1103/PhysRevB.101.026403

All these are not cited nor discussed here.

Related to this, given the importance of breaking time-reversal symmetry as discussed in this work, it appears odd that previous work on the relation between time-reversal symmetry and CISS is not cited and discussed (see in particular the work by Bart van Wees).

- Comment on reply to my comment 5): "the scope of the paper is not to map out the viable range of parameters." - the intention underlying my comment was to show whether the conclusions made in this paper still hold when the parameters are varied within reasonable bounds. Also, my suggestion was to discuss to what extent this model is predictive, i.e., whether it can predict experimental outcomes for different molecules or atomistic configurations by choosing the parameters accordingly for each one, or whether its purpose is to rationalize experimental data a posteriori by fitting the parameters to the

experimental data after the experiment has been done. Both questions have not been addressed.

- Comment on reply to my comment 7): Related to the previous point, can the author specify when saying "I assume that the Green function can be written as a Dyson equation, an assumption suggesting that the necessary conditions are met already" how to know whether this assumption (of the perturbation being small enough for the Dyson equation to be valid) is valid for a given system under certain experimental conditions? This is important for knowing whether the proposed model is predictive (in which case the validity of the Dyson equation would have to be estimated from the parameters of the model) or whether the model is meant as an a posteriori rationalization of experimental data (in which case using the Dyson equation would be justified by being able to fit the model to the experimental data).

- Comment on reply to my comment 9): Similarly, while the author indeed mentions in the text that the vibrations are assumed to be harmonic, he has not replied to the main part of this comment: "under which circumstances would one expect the harmonic approximation to hold, and can one safely assume that it will hold for the systems under study here? (in particular, for low-frequency vibrations, the harmonic approximation tends to do badly due to the large portion of the potential energy surface being sampled)." This is crucial for assessing when this model is valid.

- (Minor) comment on reply to my comment 11): The matrix notation is somewhat more clear now, but the necessity for two sets of indices which are identical, in (Gmn)mn, is unclear and the notation appears confusing.

Author's Response to Peer Review Comments:

Institutionen för Fysik och Astronomi
Dr. Jonas Fransson
Professor

Besöksadress:
Ångströmlaboratoriet,
Polacksbacken
Lägerhyddvägen 5

Postadress:
Box 516
751 21 Uppsala

Telefon:
018 471 5864

Telefax:
018 471 3524

Mobil:
070 167 9264

Hemsida:
http://www.physics.uu.se

Epost:
Jonas.Fransson@physics.uu.se

Department of Physics and Astronomy
Jonas Fransson
Professor

Visiting address:
Ångströmlaboratoriet,
Polacksbacken
Lägerhyddvägen 5

Postal address:
Box 516
SE-751 21 Uppsala
SWEDEN

Telephone:
+46 18 471 5864

Telefax:
+46 18 471 3524

Cell:
+46 70 167 9264

Web page:
http://www.physics.uu.se

Email:
Jonas.Fransson@physics.uu.se

Dear Editor,

I hereby resubmit my manuscript *Chiral induced Spin Polarized Electron Current: Origin of the Chiral Induced Spin Selectivity Effect*, for consideration for publication in the Journal of Physical Chemistry Letters.

While the reviewers are both in favor of this article, they still ask me to address a few questions which I have done in this revised manuscript. Overall, the reviewers have been very constructive and helpful, and I hope that the revised manuscript is even clearer in the presentation of the material. Below, I have responded to the raised issues in detail. All changes are high-lighted in the additional manuscript pdf.

I also want to comment on the changes of the figures. The second reviewer asked for inclusion of anharmonic effects to the vibrational modes, which I have made in this revised manuscript. The, thus, altered calculation scheme renders different numerical results which is why the plots have had to be changed. However, the main conclusion that is made in this manuscript remains unchanged by the introduction of anharmonicity, in fact, if anything including anharmonicity actually strengthens the conclusion.

Having addressed the questions raised by the reviewers and made corresponding amendments in the manuscript, I believe that my article is suitable for publication in the Journal of Physical Chemistry Letters.

Yours sincerely,

Jonas Fransson

Response to the first reviewer

Recommendation: This paper is probably publishable, but major revision is needed; I do not need to see future revisions.

- **Reviewer comment 1)** *Comments: The Author answered most of my questions in the revised manuscript.*

  However, one point has not still been satisfactorily addressed. The frequency of the vibrational modes used in most of the simulations is unreasonably small for real molecules. Such low energy vibrations can only exist in the solid state (or on a surface) and correspond to long wavelength acoustic phonons, i.e. rigid translations of the molecule. Hence, they cannot influence significantly Hamiltonian parameters at the molecular scale such as the nearest-neighbor hopping. In general, I would expect low-energy modes to have a very small electron-phonon coupling.

  Hence, before recommending publication, the Author should include simulations of the spin polarization and of the current (Figs. 1 and 3) with a reasonable frequency of the vibrations in the meV range and explicitly report in the main text this parameter.

  My response: The reviewer points out a very important question, which is something that has also confused me since I introduced the model used here. That is, why are the vibrational frequencies have to be very low, corresponding to energies in the μeV range which seems to be far below, at least, any experimentally obtained energies. Thanks to the second reviewer, I reconsidered the modeling by also including effects of anharmonicity, which is reasonable in a structure with broken inversion symmetry, such as a chiral one. The revised calculations are based on vibrational energies in the meV range, which are yet at the lower end of experimentally recorded energies, nevertheless, not out of the feasible range and such energies have also been measured. I, therefore, hope that I have answered the question raised concerning the vibrational energies.

Response to the second reviewer

This paper may be publishable, but major revision is needed; I would like to be invited to review any future revision.

- **Reviewer comment 1)** *The manuscript has been improved. There are still open questions and issues, in particular concerning the novelty of this work compared to previous discussions in the literature and the range of validity of the proposed model:*

  My response:

- **Reviewer comment 2)** *The (potential) importance of dissipation in CISS was, contrary to what the author is stating, brought up and discussed as*

an essential component for the CISS mechanism in several instances in the literature, e.g.:

https://doi.org/10.1002/adma.202106629
https://doi.org/10.1021/acs.jpclett.0c00474
https://doi.org/10.1103/PhysRevB.104.024430
https://doi.org/10.1021/acs.jpclett.8b02196
https://doi.org/10.1021/acs.jpclett.3c02546
https://doi.org/10.1103/PhysRevB.101.026403

All these are not cited nor discussed here.

My response: I thank the reviewer for providing references. However, except for https://doi.org/10.1103/PhysRevB.101.026403, which as also been added, all other references were already included in the revised manuscript.

Related to this, given the importance of breaking time-reversal symmetry as discussed in this work, it appears odd that previous work on the relation between time-reversal symmetry and CISS is not cited and discussed (see in particular the work by Bart van Wees).

My response: The reviewer correctly points out that I have not included many references regarding breaking time-reversal symmetry. This has been amended in the revised manuscript, however, I also notice that while this is an on-going discussion in the field there is very little tangible about how to resolve this issue.

- **Reviewer comment 3)** *Comment on reply to my comment 5): "the scope of the paper is not to map out the viable range of parameters." - the intention underlying my comment was to show whether the conclusions made in this paper still hold when the parameters are varied within reasonable bounds. Also, my suggestion was to discuss to what extent this model is predictive, i.e., whether it can predict experimental outcomes for different molecules or atomistic configurations by choosing the parameters accordingly for each one, or whether its purpose is to rationalize experimental data a posteriori by fitting the parameters to the experimental data after the experiment has been done. Both questions have not been addressed.*

  My response: I thank the reviewer for clarifying the question. First, the main set of parameters are chosen to be more or less reasonable for a realistic context with molecules in junctions. The key issue, however, is to keep the spin-orbit coupling parameters λ_i, $i = 0, 1$, (at least two and three) orders of magnitude less than the hopping rate t_0, in order to demonstrate that a large effect can be obtained without resorting to unrealistic values for those parameters. The crucial point is that electron correlations make the difference, partially by enhancing the effect of the spin-orbit coupling and partially by introducing polarizability in the system.

  Regarding the predictive power of effective modeling, this is always a delicate issue. There is certainly a predictive power concerning the phenomenology, perhaps even in terms of relating parameters with each other – should,

e.g., λ_0 be of this or that order with respect to t_0, and so forth. At best, one can say something about how the parameters should be related, however, quantitative predictions cannot be made using a very simplified model as the one I am using.

I have added to the discussion at the end of the manuscript concerning both these aspects.

- **Reviewer comment 4)** *Comment on reply to my comment 7): Related to the previous point, can the author specify when saying "I assume that the Green function can be written as a Dyson equation, an assumption suggesting that the necessary conditions are met already" how to know whether this assumption (of the perturbation being small enough for the Dyson equation to be valid) is valid for a given system under certain experimental conditions? This is important for knowing whether the proposed model is predictive (in which case the validity of the Dyson equation would have to be estimated from the parameters of the model) or whether the model is meant as an a posteriori rationalization of experimental data (in which case using the Dyson equation would be justified by being able to fit the model to the experimental data).*

  My response: Indeed, how can we tell whether the theory and experiments are connected? Typically, the modeling consideration has to be made in a perturbative manner, since the models are too complicated to allow for analytical or exact numerical solutions. Therefore, the predictive power is partially lost. However, despite the limitations of the theory, there are yet feasible and viable comments to be made that apply to the experiment, especially about phenomenological aspects. Therefore, even with a perturbative approach which allows to formulate the physics in terms of a Dyson equation contains useful information about the mechanisms that have to be present for the phenomenon to emerge. This is the perception used in the discussion in the manuscript.

- **Reviewer comment 5)** *Comment on reply to my comment 9): Similarly, while the author indeed mentions in the text that the vibrations are assumed to be harmonic, he has not replied to the main part of this comment: "under which circumstances would one expect the harmonic approximation to hold, and can one safely assume that it will hold for the systems under study here? (in particular, for low-frequency vibrations, the harmonic approximation tends to do badly due to the large portion of the potential energy surface being sampled)." This is crucial for assessing when this model is valid.*

  My response: Actually, I want to thank the reviewer for its persistence on this questions since it has forced me to reconsider the modeling and calculations. While most of the previous conclusions remain intact, inclusion of anharmonicity allows an increase of both the bare vibrational energy ω_ν as well as the associated life-time τ_{ph}. Both these increases are beneficial for the modeling reliability and applicability since the numbers are now better

on par with experimental observations. Inclusion of the anharmonic contribution leads to that I can replace the very low energies in the μeV range with energies in the meV range. This is an improvement of the modeling since it better captures realistic configurations.

I want the reviewer to observe that the slightly modified model requires updated calculations which result in plots that look different from the previous ones. It should be stressed, nonetheless, that (i) this is a natural consequence of changes in the calculations, and (ii) it is not the exact shape of the plots which is the main target, it is the effect of chirality and correlations and how this leads to an induced spin structure. This conclusion remains unchanged.

- **Reviewer comment 6)** *(Minor) comment on reply to my comment 11): The matrix notation is somewhat more clear now, but the necessity for two sets of indices which are identical, in $\{G_{mn}\}_{mn}$, is unclear and the notation appears confusing.*

  My response: I agree with the reviewer that the notation $\mathbf{G}_{mm}$ may appear odd. However, the two-index notation is necessary since $\mathbb{G}$ is an $\mathbb{M} \times \mathbb{M}$-matrix in coordinate space and to not have to switch between different notations for diagonal, $\mathbf{G}_m$, and off-diagonal, $\mathbf{G}_{mn}$, elements, which can also be confusing, I have chosen to keep one single notation for the sake of clarity.
